# Development of an Enzyme-Based Electrochemical Acetone Gas Sensor Printed on a Porous Polyimide Film

**DOI:** 10.1021/acsomega.6c01572

**Published:** 2026-05-19

**Authors:** Isao Shitanda, Kaede Hayase, Noya Loew, Hikari Watanabe, Masayuki Itagaki

**Affiliations:** † 26413 Tokyo University of Science, 2641 Yamazaki, Noda, Chiba 278-8510, Japan; ‡ Research Institute for Science and Technology, Tokyo University of Science, 2641 Yamazaki, Noda, Chiba 278-8510, Japan

## Abstract

Acetone is a key ketone body whose concentration reflects metabolic states such as fasting, exercise, and diabetes. In this study, we report an enzyme-based electrochemical acetone gas sensor fabricated by screen printing on a porous polyimide film. The porous substrate permitted gas permeation while suppressing liquid penetration, enabling stable electrochemical measurements. The working electrode was prepared using a MgO-templated carbon graft polymerized with glycidyl methacrylate (GMgOC), which provides reactive epoxy groups for enzyme immobilization. A compact sensor was constructed by sealing a mediator-containing electrolyte within a polydimethylsiloxane chamber. The sensor exhibited concentration-dependent responses to acetone gas in the range of 50–1000 ppb, covering typical human skin gas levels. The limit of detection was 75 ppb and the limit of quantification was 250 ppb, with a linear range of 50–100 ppb. This ppb-level sensitivity is considered to be associated with the adsorption and concentration of acetone within the porous GMgOC structure. These results suggest the potential applicability of this approach for noninvasive monitoring of skin acetone gas, although further validation including selectivity testing and real-sample measurements is needed.

## Graphical abstract

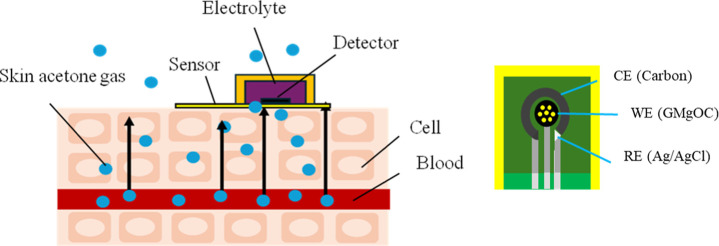

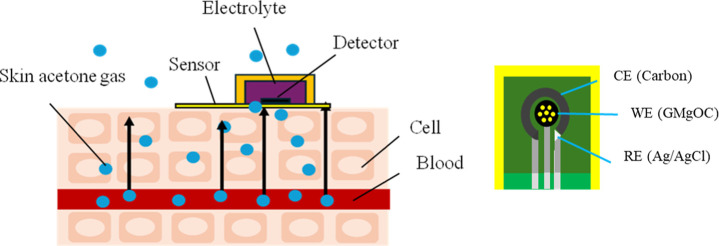

## Introduction

Exhaled breath and skin gas, types of biological gases, contain hundreds of volatile organic compounds (VOCs).^1–6^ Because the concentrations of these VOCs vary with metabolic states and disease conditions, they have received considerable attention as noninvasive biomarkers for biological monitoring and disease screening. Among VOCs, acetone is a ketone body produced through β-oxidation of fatty acid and decarboxylation of acetoacetic acid. Its production varies with the energy metabolism status and is known to increase during starvation, fasting, exercise, ketogenic diet intake, and metabolic abnormalities, such as diabetes.^7–11^

Therefore, the acetone concentration is a valuable biomarker reflecting lipid and glucose metabolisms, with potential applications in diabetes screening and evaluating dietary or fasting states. Acetone is present in the blood as well as in exhaled breath and skin gas. Breath acetone concentrations in healthy adults are reported to be approximately 200–900 ppb,^7^ while those in diabetic patients can exceed 1800 ppb.^12,13^ In contrast, acetone concentrations in skin gas are lower, ranging between 77 and 97 ppb in healthy individuals and between 171 and 205 ppb in diabetic patients.^14^

Electrochemical detection of gas-phase analytes relies on a triple-phase boundary at the interface between the gas phase, electrolyte, and electrode surface. In enzyme-based gas sensors, the analyte dissolves into the liquid electrolyte film and undergoes enzymatic reaction at the electrode, generating an electrical signal. Ensuring efficient gas–liquid–solid contact at this boundary is therefore critical for achieving high sensitivity.

Traditionally, gas chromatography–mass spectrometry (GC–MS)^15–18^ has been used to measure acetone gas concentrations. Although it offers high analytical precision, bulky equipment and complex operation make it unsuitable for real-time monitoring. In contrast, semiconductor sensors^19,20^ are compact and highly sensitive but operate at high temperatures and are susceptible to humidity and coexisting gases.

Recently, optical enzyme gas sensors (biosniffers)^7,21–24^ utilizing enzymatic reactions have emerged, enabling highly sensitive and selective measurements. However, these sensors are limited by the need for costly equipment such as high-sensitivity cameras.

The enzyme secondary alcohol dehydrogenase (s-ADH) catalyzes the reversible conversion between acetone and 2-propanol by using NADH as a coenzyme. Because s-ADH exhibits preferential activity toward secondary alcohols and ketones, it provides a basis for selective acetone detection in complex gas matrices, although direct selectivity studies against potential interferents were not performed in the present work.

While numerous wearable electrochemical sensors for liquid-phase biomarkers (glucose, lactate, uric acid) have been developed,^25,26^ relatively few reports address wearable gas-phase biosensors. A key challenge is the prevention of liquid electrolyte leakage while maintaining efficient gas permeation to the sensing electrodes. The porous polyimide film employed in the present study addresses this challenge by providing a gas-permeable, liquid-blocking substrate. A schematic of the proposed wearable configuration is provided in the Supporting Information (Figure S1).

One approach to improving sensitivity is the use of electrodes with a high specific surface area. Shitanda et al. analyzed a cathode electrode composed of MgO-molded carbon (MgOC, CNovel, Toyo Carbon), a porous carbon material templated on MgO. They reported that the porous electrode with a branched pore structure fabricated using MgOC exhibited higher current values than electrodes without branched pores.^27^

Furthermore, GMgOC, produced by graft polymerizing glycidyl methacrylate (GMA) onto the MgOC surface, enables stable enzyme immobilization within its pores. The epoxy groups of GMA react with amino groups of the enzyme, facilitating its attachment to the electrode surface.^28^

Previous studies have developed enzyme-based gas sensors for acetaldehyde and ethanol using gel electrolytes in electrochemical sensing systems that utilize enzymatic reactions.^29,30^ These studies have demonstrated the capability of these sensors to selectively detect low-concentration VOCs.

Building on these previous reports, the present work combines (i) a porous polyimide film that allows only gas permeation while blocking liquid, (ii) GMgOC with high specific surface area for analyte concentration, and (iii) screen printing for facile, scalable fabrication. This combination enables parts per billion-level acetone detection within a compact device.

Due to the enzymatic reaction of secondary alcohol dehydrogenase (s-ADH), the biosensor is expected to provide a selective response based on the substrate specificity of s-ADH. In this study, acetone was selected as the target molecule. An electrochemical biosensor capable of detecting acetone gas at skin gas levels was developed by using an electrode printed on a porous polyimide film and s-ADH. The objective of this study was to achieve sensitive detection of low-concentration acetone gas at the parts per billion level by combining a porous polyimide film that allowed only gas permeation with GMgOC, suitable for enzyme immobilization.

## Materials and Methods

### Materials

The porous polyimide film PIM-1000N (thickness: 40 μm, porosity: 70%, permeability: 40.4 s/100 mL, and an average pore diameter of approximately 1 μm) was purchased from Tokyo Ohka Kogyo Co., Ltd. (Figure S2). GMgOC was prepared by grafting GMA onto MgO template carbon CNovel© (average pore size of MgOC = 100 nm) purchased from Toyo Tanso Co., Ltd., after its electron beam irradiation (100 kGy) at Japan Irradiation Service Co., Ltd. Polyvinylidene fluoride–hexamethylene copolymer (PVdF: KF Polymer L#9305 5% in NMP) was procured from Kureha. 1-Methyl-2-pyrrolidone (NMP), 1-methoxy PMS, and reduced nicotinamide adenine dinucleotide (NADH) were obtained from Fujifilm Wako Pure Chemical Industries. The polydimethylsiloxane (PDMS) chamber was fabricated by mixing liquid silicone rubber KE-106, obtained from Shin-Etsu Chemical Co., Ltd., with the curing agent CAT-RG. Secondary alcohol dehydrogenase s-ADH (Chiral Screen OH E001 0.45 U/mg) was obtained from Daicel Corporation. The gas generator and acetone permeation tube (P-151-H) were purchased from Gastec Corporation, and the gas flow rate was regulated using a mass flow controller purchased from Coflock Corporation.

### Characterization of GMgOC

The morphology of MgOC and GMgOC was characterized by field emission scanning electron microscopy (FE-SEM; Hitachi SU-8010, accelerating voltage of 5.0 kV, working distance of 7.0 mm). FE-SEM images confirmed the characteristic porous, interconnected carbon framework of MgOC and showed that GMA grafting did not substantially alter the macroscopic pore structure while adding a surface coating layer visible as increased surface roughness.

GMA grafting on MgOC was confirmed by FT-IR spectroscopy. The FT-IR spectrum of GMgOC showed characteristic absorption bands at approximately 3000 cm^–1^ (C–H stretching of the GMA alkyl chain), 1750 cm^–1^ (C=O stretching of the ester group in GMA), 1100 cm^–1^ (C–O–C stretching), and 910 cm^–1^ (epoxy ring vibration), which are absent in unmodified MgOC. The presence of the epoxy ring band at 910 cm^–1^ confirms successful GMA grafting on the MgOC surface.

Raman spectroscopy of GMgOC was not performed in this study. Raman characterization—particularly the D-band (∼1350 cm^–1^) and G-band (∼1580 cm^–1^) analysis to assess the graphitization degree—is planned as a topic for future investigation.

X-ray photoelectron spectroscopy (XPS) C 1s analysis of GMgOC confirmed GMA grafting. The C 1s spectrum was deconvoluted into four components: 284.7 eV (C–C/C–H), 285.4 eV (C–C=O), 287.1 eV (C–O–C, ether/epoxide), and 289.3 eV (O–C=O, ester). The ester peak at 289.3 eV and the ether/epoxide component at 287.1 eV are characteristic of the GMA side chain and epoxide group, respectively, consistent with successful GMA grafting.

### Fabrication of the Printed Acetone Gas Biosensor

A three-electrode system was employed for fabricating the sensor. The GMgOC ink for the working electrode was prepared by mixing 200 mg of GMgOC powder, 1.4 mL of NMP, and 0.8 mL of PVdF using a rotary-orbital mixer (mixing speed: 2000 rpm for 1 min; deforming speed: 2200 rpm for 1 min). The Ag/AgCl ink for the reference electrode was prepared by initially dissolving silver nitrate in pure water and mixing the resulting solution with potassium chloride to precipitate AgCl. This precipitate was subsequently recovered, washed, and finally redispersed for storage. Furthermore, it was dried at 135 °C to yield the AgCl powder. This powder was then mixed with the Ag ink at a 1:10 weight ratio to produce the Ag/AgCl ink. The electrodes were fabricated by screen printing using a NEWLONG LS-150TV screen printer. Printing was performed on a porous polyimide film in the sequence illustrated in Figure 1a, and a schematic of the prepared electrode is shown in Figure 1b.

**Figure 1:**
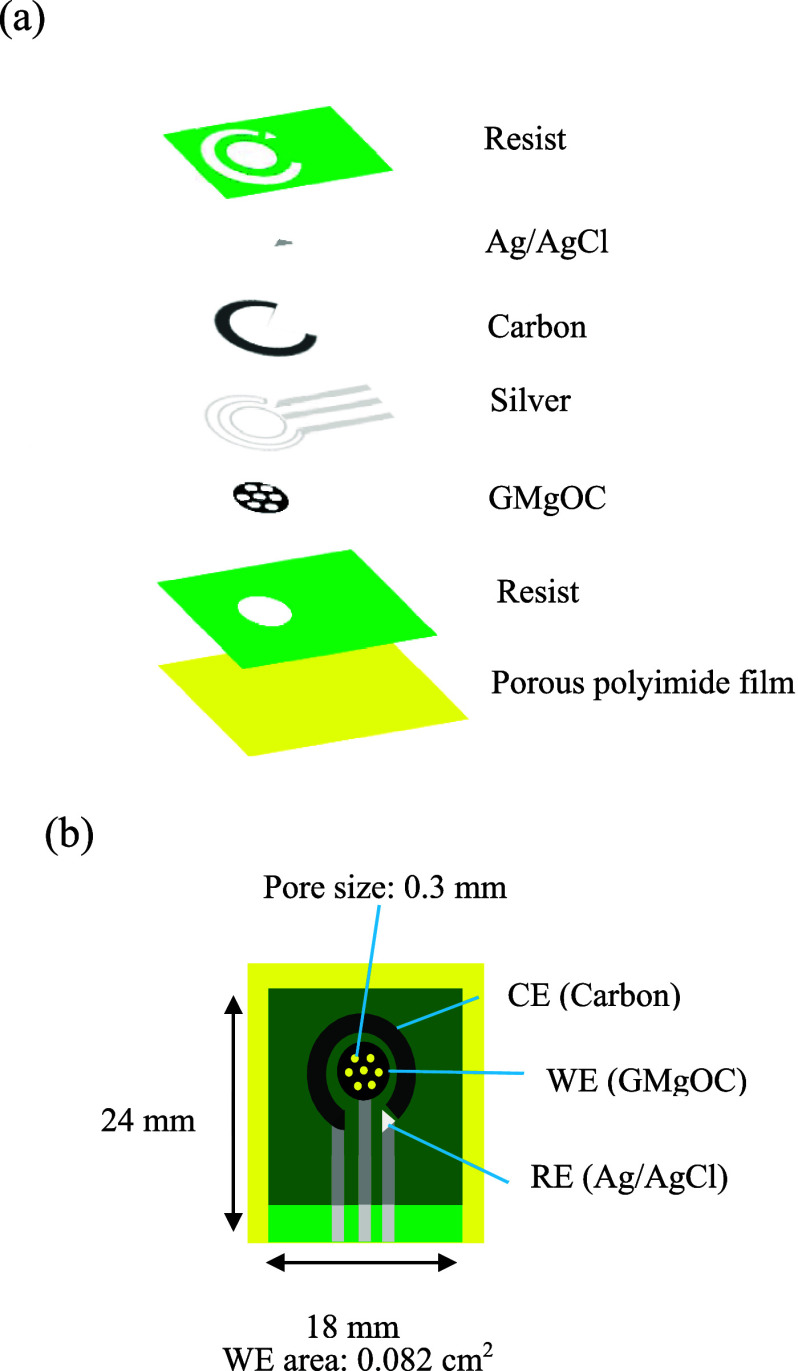
Schematic of the printed gas sensor: (a) printing sequence (from bottom to top): resist ink, GMgOC ink, silver ink, carbon ink, silver/silver chloride ink, and resist ink. (b) Schematic of a printed sensor with the working electrode, counter electrode, and reference electrode.

The screen-printing conditions used for the GMgOC working electrode are summarized in Table 1. These parameters were optimized to achieve a uniform ink deposition on the porous polyimide substrate. The drying conditions for each ink layer are given in Table 2.

**Table 1:** Screen-Printing Conditions for GMgOC Electrode Fabrication

parameter	value
squeegee angle	75°
clearance	1.00 mm
work thickness	0.04 mm
coating amount	132 mm
printing amount	62 mm
coating speed	30 mm/s
printing speed	30 mm/s

**Table 2:** Drying Conditions for Each Ink Layer

ink layer	temperature (°C)	duration
resist ink	180	120 min
GMgOC ink	60	overnight
silver ink	130	60 min
carbon ink	130	60 min
Ag/AgCl ink	130	60 min

Specifically, a 10 μL s-ADH solution dissolved in 10 mM phosphate buffer (pH = 7.0) was dispensed onto the working electrode. The enzyme was immobilized on the working electrode via drying under reduced pressure (−0.1 MPa) for 1.5 h. The chamber was fabricated by pouring PDMS into a mold and curing it at 80 °C for 1 h. This chamber was then attached to the enzyme-modified electrode using a double-sided tape (3M). Prior to measurement, a 0.5 mL measurement solution—0.1 M phosphate buffer (pH = 7.0) containing 10 mM 1-methoxy PMS and 1 mM NADH—was injected into the chamber using a syringe.

### Gas Measurement System

A permeator and permeation tube were used to generate the acetone standard gas. The tube was placed inside the permeator, which was connected to an external nitrogen gas cylinder. Nitrogen gas was supplied at 0.2 L/min for 24 h until the acetone diffusion rate stabilized (Figure 2a). The permeator was connected to the measurement cell with the electrode positioned at the top of the cell (Figure 2b). The acetone gas concentration was controlled by adjusting the nitrogen gas flow rate and permeator temperature, while the flow rate was regulated with a mass flow controller. Electrochemical measurements were performed by using a potentiostat (PalmSens).

**Figure 2:**
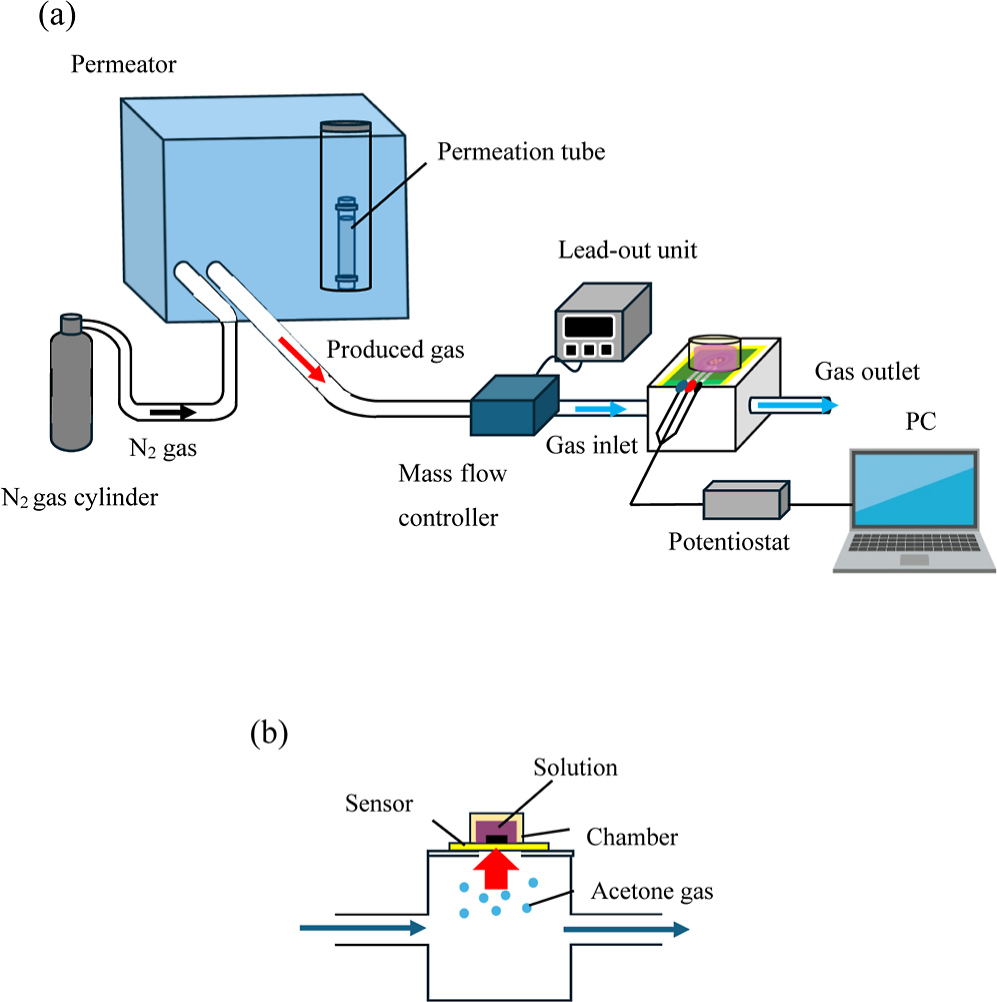
Schematic of the gas measurement workflow: (a) overall measurement setup, including the permeater, acetone gas, and gas sensor. (b) Cross-sectional view of the sensor with the measurement solution and gas supply chamber.

The acetone standard gas was generated using a Permeater PD-1B (Gastec Corporation) equipped with a P-151-H permeation tube containing liquid acetone. The acetone permeation rate was 78.2 ng/min/cm at the calibrated temperature. The gas concentration was calculated using the formula *C* = *K* × *P*_r_ × *L*/*F*, where *K* is a unit conversion constant, *P*_r_ is the permeation rate (ng/min/cm), *L* is the tube length (cm), and *F* is the carrier gas flow rate (mL/min). Details of the concentration calculation and the relationship between N_2_ flow rate and acetone concentration are provided in the Supporting Information (Table S1; see also Figure S3 for the schematic of the apparatus).

### Electrochemical Evaluation

Chronoamperometry measurements were performed by first applying a potential of −0.15 V for 60 s, followed by −0.4 V. Nitrogen gas was used to achieve acetone concentrations of 50, 80, 100, 200, 300, 500, and 1000 ppb. Each measurement lasted 500 s, performed at a gas flow rate of 30 mL/min.

## Results and Discussion

### Characterization of GMgOC

FE-SEM images of MgOC and GMgOC revealed a porous interconnected carbon framework with a hierarchical pore structure. After GMA grafting, the surface morphology was largely retained, consistent with the expectation that graft polymerization occurs at the surface without collapse of the pore architecture. No significant blockage of the pores was observed, suggesting that gas and liquid diffusion pathways remain functional after modification (Figure S4).

The FT-IR spectrum of GMgOC showed prominent absorption bands at ∼3000 cm^–1^ (C–H stretching), ∼1750 cm^–1^ (C=O ester), ∼1100 cm^–1^ (C–O–C), and ∼910 cm^–1^ (epoxy ring vibration), which are absent in unmodified MgOC (Figure S5). These bands are consistent with the presence of the GMA side chain and confirm successful GMA grafting onto the MgOC surface.

XPS C 1s analysis of GMgOC yielded four deconvoluted peaks: 284.7 eV (C–C/C–H), 285.4 eV (C–C=O), 287.1 eV (C–O–C/epoxide), and 289.3 eV (O–C=O/ester) (Figure S6). The ester (289.3 eV) and ether/epoxide (287.1 eV) components are characteristic of poly-GMA and confirm GMA grafting, consistent with the FT-IR results.

### Optimization of the Enzyme Modification Amount

The prepared sensor had an enzyme-type electrode in which s-ADH was immobilized on the working electrode through bonding between the epoxy group of GMgOC and the amino group of s-ADH. NADH in solution acted as a coenzyme, and 1-methoxy-PMS functioned as an electron mediator (Figure 3a). In this reaction system, acetone was reduced by s-ADH, and the generated NAD^+^ was detected as an electrical response via an electrode reaction.

**Figure 3:**
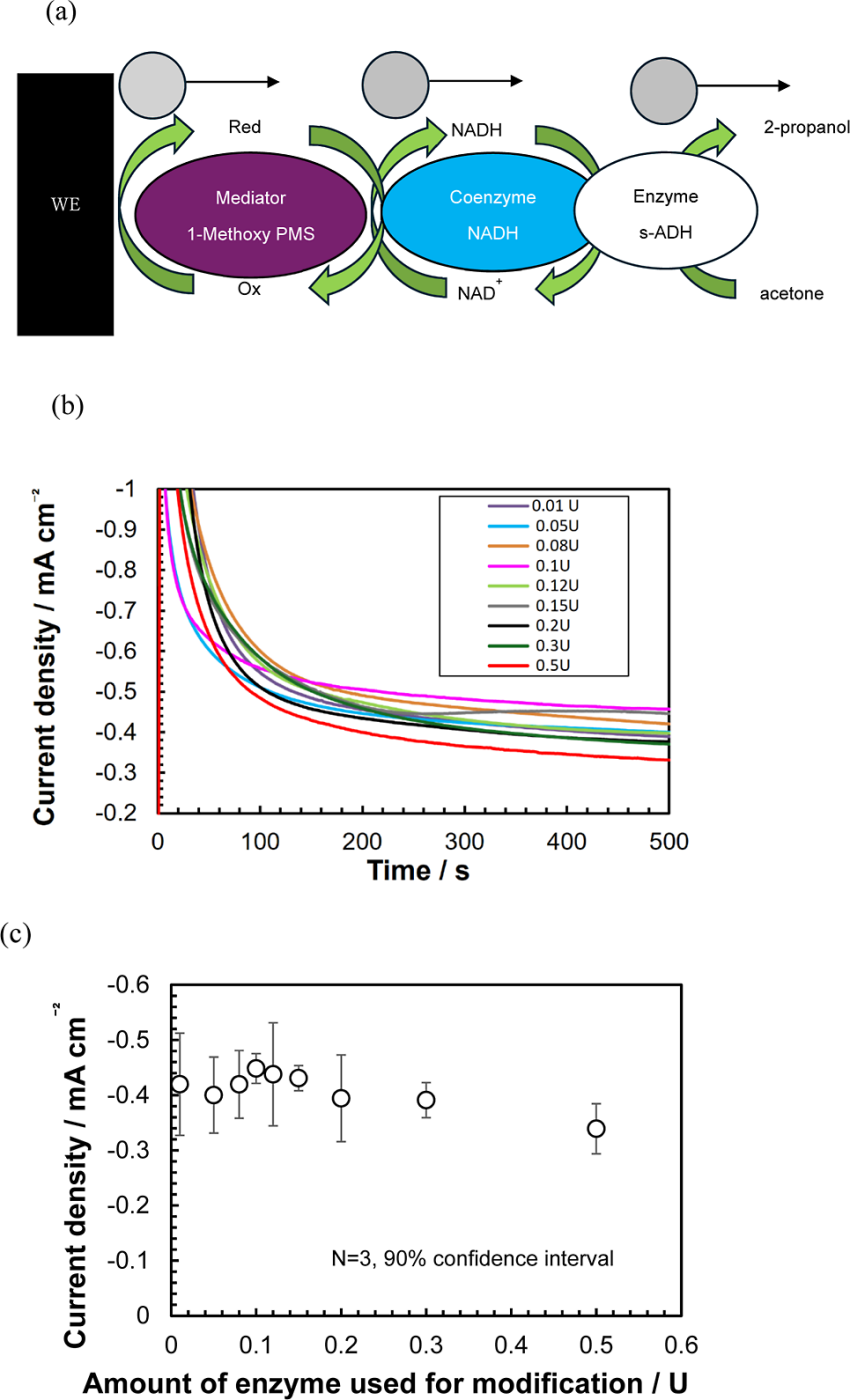
(a) Sensing mechanism of the prepared sensor. (b) Chronoamperogram of the proposed sensors whose electrodes are modified using varying enzyme levels (electrode: printed three-electrode system; measurement solution: 0.1 M phosphate buffer (pH = 7.0) containing 10 mM 1-methoxy PMS and 1 mM NADH; measurement potential: −0.4 V; measurement time: 500 s, acetone gas concentration: 1000 ppb, and gas flow rate: 30 mL/min). (c) Current density measured after 500 s by the proposed sensors whose electrodes are modified using varying enzyme levels (number of measurements: 3; 90% confidence interval).

After permeating through the porous polyimide substrate, acetone vapor dissolves into the electrolyte solution within the PDMS chamber. The dissolved acetone then undergoes enzymatic reduction by s-ADH, consuming NADH and producing NAD^+^ and 2-propanol. The oxidation of 1-methoxy PMS (reduced form) by the electrode at −0.4 V regenerates the mediator and generates the amperometric signal. This reaction cascade links gas-phase acetone concentration to the measurable current.

The effect of enzyme modification on the electrical response was first investigated (Figure 3b). The highest current was observed for the electrode modified with 0.1 U of the enzyme (Figure 3c). At lower enzyme levels, the response decreased because the reaction between acetone and s-ADH was the rate-limiting step. Furthermore, electrode modification with an excessive amount of enzyme reduced the response, likely because of the increased electron transfer resistance.

1-Methoxy PMS: 1-methoxy-5-methylphenadinimethoxysulfate; s-ADH: secondary alcohol dehydrogenase; and NADH: reduced nicotinamide adenine dinucleotide.

### Acetone Gas Sensing

The sensor response to acetone gas was evaluated across various concentrations (50–1000 ppb) (Figure 4a). Compared to the background current in the absence of acetone, the current density increased in the presence of acetone gas. This response is consistent with the enzymatic reaction coupled with mediator-facilitated electron transfer.

**Figure 4:**
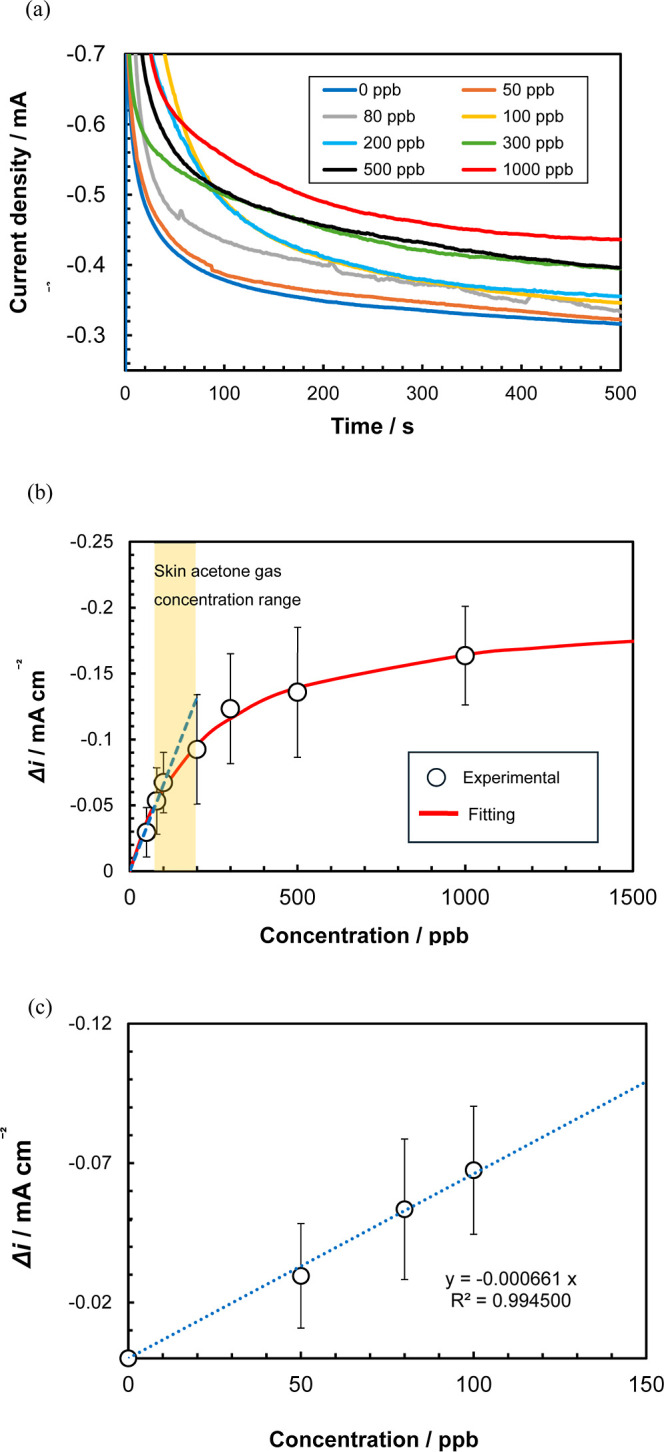
(a) Chronoamperograms of the sensor at various acetone gas concentrations (electrode: printed three-electrode system; measurement solution: 0.1 M phosphate buffer (pH = 7.0) containing 10 mM 1-methoxy PMS and 1 mM NADH; measurement potential: −0.4 V, measurement time: 500 s, acetone gas concentrations: 0, 50, 80, 100, 200, 300, 500, and 1000 ppb; and gas flow rate: 30 mL/min). (b) Calibration curve plotting the difference in the current density relative to the background current in the absence of acetone (three measurements; 90% confidence interval). (c) Slope of the calibration curve in the low acetone concentration range (50–100 ppb).

Figure 4b shows the change in the current density relative to the background current measured over 500 s in the absence of acetone against the acetone concentration. A correlation between the current density and acetone concentration was observed in the range of 50–1000 ppb, consistent with the adsorption and concentration of acetone gas within the GMgOC pores.^29^ Furthermore, linearity was achieved between 50 and 100 ppb (Figure 4c). The detection limit was 75 ppb, and the quantification limit was 250 ppb, both calculated based on the standard deviation of the background current.

The limit of detection (LOD = 75 ppb) and limit of quantification (LOQ = 250 ppb) were calculated as LOD = 3σ/S and LOQ = 10σ/S, respectively, where σ is the standard deviation of the background current (three replicate measurements at zero acetone) and S is the slope of the linear calibration curve (0.0028 mA cm^–2^ ppb^–1^, *R* ^2^ ≥ 0.99).

Considering that human skin acetone gas concentrations range from 77 to 97 ppb in healthy individuals and from 171 to 205 ppb in diabetic patients,^14^ these results suggest that the fabricated sensor can detect acetone in skin gas. The Michaelis–Menten analysis yielded a *K*_m_ of 217 ppb and a maximum current density *i*_max_ of −0.20 mA cm^–2^.

The apparent *K*_m_ of s-ADH in solution has been reported as ∼0.33 mM (approximately equivalent to ∼19,193 ppb as a rough phase-to-phase comparison in the gas phase using Henry’s law-based conversion described in the Supporting Information; Figure S7). The gas-phase *K*_m_ obtained in this study (217 ppb) is approximately 88-fold lower, suggesting possible local enrichment of acetone within the GMgOC pores, which may increase the effective concentration at the enzyme. This enrichment effect is consistent with the high specific surface area and porous structure of GMgOC; however, the precise mechanism of analyte concentration requires further investigation.

A preliminary assessment of short-term stability was conducted by observing sensor response consistency during the measurement session; however, systematic stability testing over extended periods was not performed in this study. Long-term stability evaluation, including measurement of response retention after repeated use, is planned for future work.

The intersensor fabrication reproducibility was not systematically evaluated in the present study, as a sufficient number of independently fabricated sensors was not tested under identical conditions. Quantitative assessment of reproducibility (expressed as relative standard deviation, RSD) is planned for future studies.

The selectivity of the present sensor was not directly evaluated by exposing it to potential interferents, such as ethanol, 2-propanol, or acetaldehyde. The substrate specificity of s-ADH toward secondary alcohols and ketones suggests that cross-reactivity toward primary alcohols such as ethanol may be low; however, this hypothesis has not been experimentally confirmed in the present work. Systematic selectivity studies are planned as future work, following the approach of Chien et al.^22^

In this study, all measurements were performed by using nitrogen as the background carrier gas. The performance of the sensor under air or under conditions containing humidity and common atmospheric interferents was not evaluated. Such testing is necessary for practical applications and is planned as future work.

The power consumption of the present sensor was not quantitatively determined in this study. Based on the chronoamperometric conditions (applied potential: −0.4 V, typical current density: ∼0.1 mA cm^–2^, electrode area: ∼0.09 cm^2^), the instantaneous power is estimated to be on the order of a few microwatts, which is low compared to semiconductor sensors that require elevated operating temperatures. Quantitative power measurements are planned as future work.

The response time and recovery time of the sensor were not systematically measured in this study. Furthermore, the memory effect—residual signal from previous acetone exposure—was not evaluated. These parameters are important for practical sensor deployment and will be addressed in future work.

The liquid-blocking performance of the porous polyimide film was not quantitatively assessed by contact angle measurements or liquid penetration tests in this study. The film manufacturer specifies a gas permeability of 40.4 s/100 mL and a pore size of ∼1 μm (Table S2), which are expected to prevent liquid electrolytes from leaking through the substrate; however, experimental verification under realistic sensor operating conditions is planned as future work. A schematic illustration of a possible wearable device configuration is provided in Figure S1 for reference.

Real sample measurements using actual skin gas from human subjects and comparison with GC–MS reference values were not performed in the present study. Such validation is essential for confirming the practical utility of the sensor and will be conducted in future work.

### Future Applications

The present sensing strategy combines gas-permeable porous polyimide with GMgOC, which can adsorb and concentrate analytes in its pores, enabling ppb-level acetone detection. The feasibility of performing skin gas measurement using this device configuration is demonstrated in Figure S1. Because gas permeation, preconcentration, and electrochemical detection steps can be independently designed, this platform is adaptable to other VOCs by selecting appropriate enzyme–mediator systems. These features highlight its potential as a compact and selective sensing platform for noninvasive gas monitoring.

Future work will focus on (1) systematic evaluation of sensor selectivity against common skin gas components (ethanol, 2-propanol, acetaldehyde) following established protocols;^23^ (2) long-term stability testing and reproducibility characterization (RSD across independently fabricated sensors); (3) evaluation of sensor performance under realistic humidity and atmospheric conditions; (4) real-sample testing using actual exhaled breath or skin gas with GC–MS validation; (5) power consumption measurements and miniaturized potentiostat integration for wearable deployment; and (6) Raman and electrochemical impedance spectroscopy characterization of the electrode interface.

## Conclusions

In this study, a printed acetone gas sensor was fabricated by using GMgOC, which was modified by graft polymerization with epoxy-functionalized poly-GMA. The epoxy groups of GMA are expected to react with the amino groups of the enzyme, enabling stable attachment of s-ADH to the electrode surface. Furthermore, the porous polyimide substrate of the sensor was designed to allow gas permeation while suppressing the solution penetration. Electrochemical evaluation demonstrated that the sensor responded to acetone concentrations ranging from 50 to 1000 ppb, covering typical skin acetone levels. This response is consistent with the adsorption and concentration of acetone gas within the GMgOC pores.

The sensor achieved a limit of detection of 75 ppb and a limit of quantification of 250 ppb (both derived from 3σ/S and 10σ/S criteria, respectively), with a calibration slope of 0.0028 mA cm^–2^ ppb^–1^ (*R* ^2^ ≥ 0.99) in the linear range of 50–100 ppb. The gas-phase apparent *K*_m_ of 217 ppb is approximately 88-fold lower than the reported solution-phase *K*_m_ (∼0.33 mM, equivalent to ∼19,193 ppb), suggesting possible analyte enrichment in the porous electrode structure.

Material characterization by FT-IR, XPS, and FE-SEM confirmed the successful GMA grafting on MgOC. These results demonstrate that the sensor can be fabricated via screen printing and shows sensitivity to acetone at concentrations relevant to skin gas monitoring. By modification of the enzyme or mediator, this platform may be adaptable to other low-concentration VOCs in skin gas.

The present work constitutes a proof-of-concept demonstration. Key aspects, including selectivity, long-term stability, reproducibility, real-sample validation, and wearable deployment, have not yet been evaluated and will be addressed in future studies. Nonetheless, these results provide a promising basis for developing a compact screen-printed gas biosensor for metabolic monitoring applications.

## Supplementary Material



## Notes

Schematic of the proposed wearable sensor configuration (Figure S1); scanning electron micrograph of the porous polyimide film PIM-1000N surface (Figure S2); schematic of the Permeater PD-1B apparatus and acetone permeation tube P-151-H (Figure S3); FE-SEM images of MgOC and GMgOC (Figure S4); FT-IR spectra of MgOC and GMgOC (Figure S5); XPS C 1s spectrum of GMgOC with peak deconvolution (Figure S6); solution-phase calibration curve of s-ADH (Figure S7); and acetone gas concentrations and N_2_ dilution flow rates (Table S1) (PDF)

## Abbreviations

MgOC: MgO-molded carbon
VOCs: volatile organic compounds
GMA: glycidyl methacrylate
NADH: reduced nicotinamide adenine dinucleotide
NMP: 1-methyl-2-pyrrolidone
PDMS: polydimethylsiloxane
