# Supplementary material for: Development of an Enzyme-Based Electrochemical Acetone Gas Sensor Printed on a Porous Polyimide Film
Source: ACS Omega. 2026 May 19;11(21):31398–404. doi: 10.1021/acsomega.6c01572 (PMC13234890; doi:10.1021/acsomega.6c01572)
Supplement: Supplementary file 1 [file ao6c01572_si_001.pdf]

Supporting Information

DEVELOPMENT OF AN ENZYME-BASED ELECTROCHEMICAL ACETONE GAS SENSOR PRINTED ON A POROUS POLYIMIDE FILM

Isao Shitanda[a,b, ‡,*], Kaede Hayase[a, ‡], Noya Loew[a], Hikari Watanabe[a],

and Masayuki Itagaki[a,b]

[a] *Tokyo University of Science, 2641 Yamazaki, Noda, Chiba 278-8510, Japan*

[b] Research Institute for Science and Technology, Tokyo University of Science, 2641 Yamazaki, Noda, Chiba 278-8510, Japan

Table of Contents Section

Section S1. Wearable Sensor Configuration (Figure S1)
Section S2. Porous Polyimide Film PIM-1000N (Figure S2)
Section S3. Acetone Standard Gas Generation (Figure S3, Table S1)
Section S4. FE-SEM Characterization of GMgOC (Figure S4)
Section S5. FT-IR Characterization of GMgOC (Figure S5)
Section S6. XPS Characterization of GMgOC (Figure S6)
Section S7. Solution Calibration and Enrichment Factor (Figure S7, Table S1)
Section S8. Selection Rationale for s-ADH (Table S2)
Section S9. Calculation of LOD and LOQ
Section S10. Sensor Selectivity Section
Section S11. Scope of the Present Study and Future Work

Section S1. Wearable Sensor Configuration

Figure S1 shows a schematic illustration of the proposed wearable sensor configuration attached to the human body. The sensor is designed for direct skin contact, enabling noninvasive, real-time monitoring of skin gas acetone without sample collection.

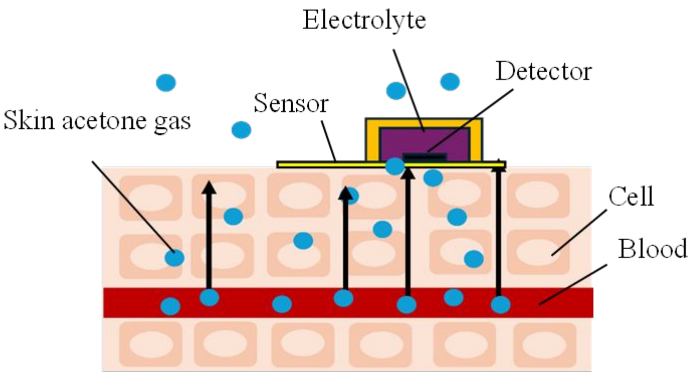

Figure S1. Schematic of the proposed wearable sensor attached to the human body for noninvasive skin gas acetone monitoring.

The wearable configuration leverages the porous polyimide substrate to allow gas permeation from the skin surface while preventing sweat ingress into the electrolyte chamber. The PDMS chamber seals the mediator-containing electrolyte and maintains a stable electrochemical environment. This design enables long-term wearable operation without the need for external gas-collection equipment.

Section S2. Porous Polyimide Film (PIM-1000N)

S2.1 Porous Polyimide Film (PIM-1000N)

Polyimide films are widely used in electronics materials due to their excellent thermal stability, electrical insulation, and mechanical strength. For the substrate of a wearable gas sensor, key requirements include high gas permeability, compatibility with screen-printing processes, and the ability to block liquid ingress to protect the enclosed electrolyte. The porous polyimide film PIM-1000N (Tokyo Ohka Kogyo Co., Ltd.)[1] satisfies all of these requirements: it has a well-defined pore diameter of ~1 μm that allows gas diffusion while preventing liquid penetration, and its smooth surface is compatible with screen-printing.

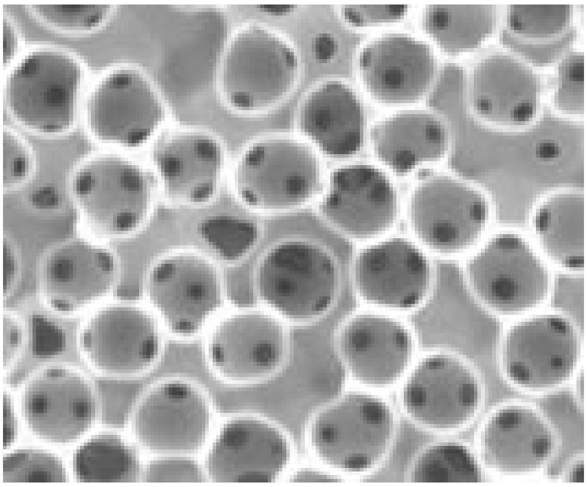

Figure S2. Scanning electron micrograph of the porous polyimide film PIM-1000N surface, showing the uniform pore structure (~1 μm diameter)[1].

Section S3. Acetone Standard Gas Generation

Acetone standard gas was generated using a Permeater PD-1B (Gastec Corporation)[2] equipped with an acetone permeation tube P-151-H (Gastec Corporation). The permeater temperature was set to 35 °C. Nitrogen gas (diluent) was supplied at a flow rate of 0.2 L min^{-1} for 24 h until the acetone diffusion rate from the permeation tube stabilized. After stabilization, the target acetone concentration was achieved by adjusting the nitrogen diluent flow rate using a mass flow controller (Coflock Corporation).

The acetone gas concentration C (ppm) was calculated using:

$$C = (K \times P_r \times L) / F \qquad \text{(S1)}$$

where K is a unit-conversion coefficient (K = 0.421 L g^{-1} at 35 °C), P_r is the permeation rate (78.2 ng min^{-1} cm^{-1} at 35 °C), L is the effective tube length (cm), F is the diluent gas flow rate (mL min^{-1}), M is the molecular weight of acetone (58.08 g mol^{-1}). Table S1 summarizes the N_2 flow rates used for each target concentration.

Table S1. Acetone gas concentrations and corresponding N_2 dilution flow rates (permeator temperature: 35 °C, permeation rate P_r = 78.2 ng min^{-1} cm^{-1}).

Acetone concentration (ppb)	N_2 flow rate (L min^{-1})
50	6.6
80	4.1
100	3.3
200	1.7
300	1.1
500	0.66
1000	0.33

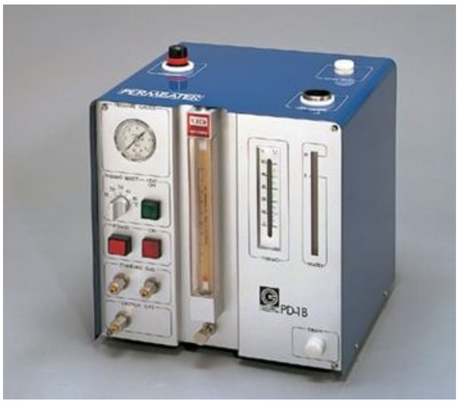

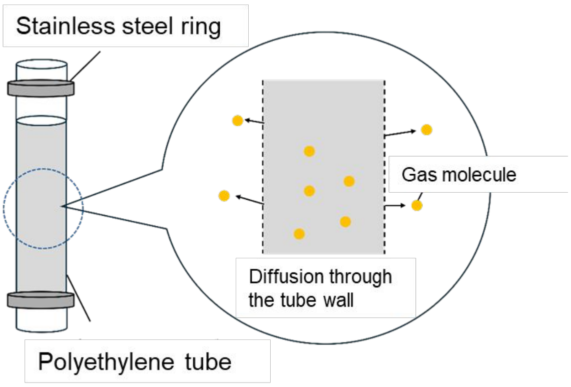

Figure S3. Schematic diagram of the Permeater PD-1B (Gastec Corporation) and acetone permeation tube P-151-H used for standard gas generation[2].

All measurements in this study were performed using nitrogen (N_2) as the diluent and background gas. The effect of background gas composition (e.g., ambient air, oxygen, water vapor, CO_2) on sensor performance was not evaluated and is acknowledged as a limitation. Performance under ambient air conditions is planned as future work.

Section S4. Characterization of GMgOC

This section presents the material characterization data for GMgOC (MgO-templated carbon graft-polymerized with glycidyl methacrylate) used in the present study[3,4].
The surface morphology of MgOC and GMgOC was characterized by field-emission scanning electron microscopy (FE-SEM) at an accelerating voltage of 5.0 kV and a working distance of 7.0 mm. Figure S4 shows the FE-SEM image of GMgOC. The image reveals a porous, interconnected carbon network derived from the MgO template, with the hierarchical pore structure (average pore diameter ~100 nm) clearly preserved after GMA graft polymerization. This porous architecture provides a large electroactive surface area for enzyme immobilization and analyte preconcentration.

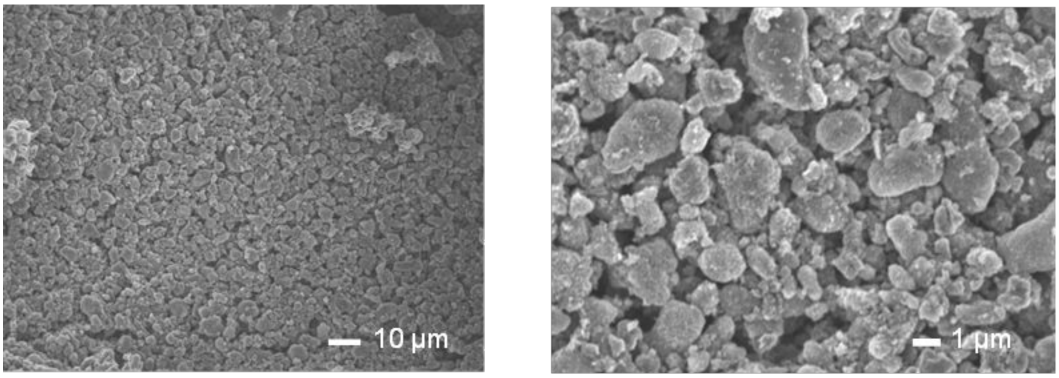

Figure S4. FE-SEM image of GMgOC (accelerating voltage: 5.0 kV, working distance: 7.0 mm). The porous, interconnected carbon network derived from the MgO template is clearly visible.

Section S5. FT-IR Characterization of GMgOC

FT-IR measurements were carried out using a Fourier-transform infrared spectrophotometer (FT/IR-6000, JASCO Corporation) over the wavenumber range 3500–500 cm^{-1} (64 accumulations, 25 °C, ambient atmosphere). Figure S5 shows the FT-IR spectra of MgOC and GMgOC.

Compared to unmodified MgOC, the GMgOC spectrum shows three additional absorption bands: approximately 3000 cm^{-1} (C–H stretching of the GMA alkyl chain), ~1750 cm^{-1} (C=O stretching of the ester group), and ~1100 cm^{-1} (C–O–C stretching of the ester/ether linkage). These bands confirm the successful introduction of poly(GMA) chains onto the MgOC surface by electron-beam-initiated graft polymerization[5]. Importantly, after s-ADH immobilization, the epoxide ring-stretching band at ~910 cm^{-1} was not detected in the GMgOC spectrum after enzyme immobilization, indicating possible ring-opening of the epoxide groups. This observation is consistent with the proposed enzyme immobilization chemistry, supporting but not directly proving covalent attachment of s-ADH.

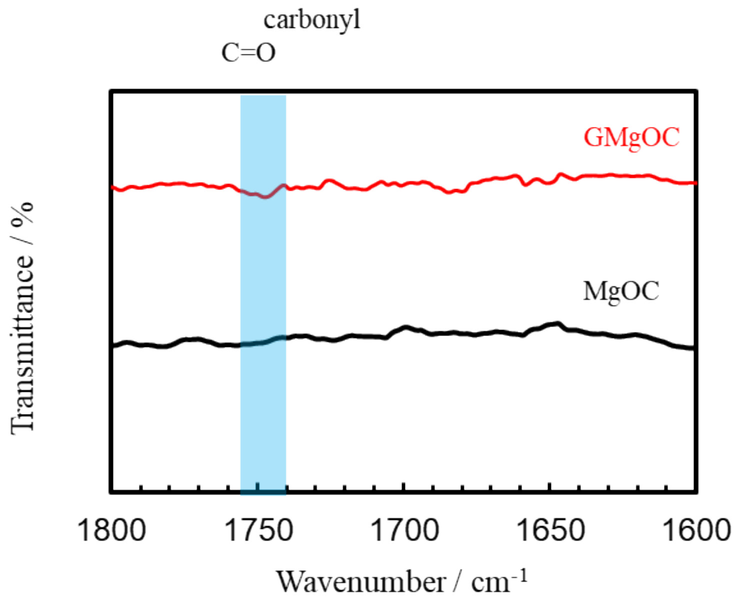

Figure S5. FT-IR spectra of MgOC (black) and GMgOC (red). New absorption bands at ~3000, ~1750, and ~1100 cm^{-1} in GMgOC confirm GMA graft polymerization. The absence of the epoxide band at ~910 cm^{-1} after enzyme immobilization is consistent with the proposed enzyme immobilization chemistry.

Section S6. XPS Characterization of GMgOC

XPS measurements were performed using an X-ray photoelectron spectrometer (AXIS Nova, Shimadzu Corporation) with Al Kα radiation (excitation energy: 1487 eV, anode

voltage: 15 kV, emission current: 10 mA). Figure S6 shows the C1s XPS spectrum of GMgOC with peak deconvolution.

The C1s spectrum of GMgOC was deconvoluted into four components: 284.7 eV (C–C/C–H, main carbon backbone), 285.4 eV (C–C=O, α-carbon to carbonyl), 287.1 eV (C–O–C-containing species), and 289.3 eV (O=C–O, ester carbonyl carbon). The 285.4, 287.1, and 289.3 eV components are consistent with the ester and epoxide functionalities introduced by GMA graft polymerization, and were absent or negligible in the spectrum of unmodified MgOC. These results confirm successful GMA grafting on the MgOC surface. BET surface area measurements were not performed in this study; characterization of the pore structure is based on FE-SEM observations and the established MgO-template synthesis procedure. BET analysis is planned as future work.

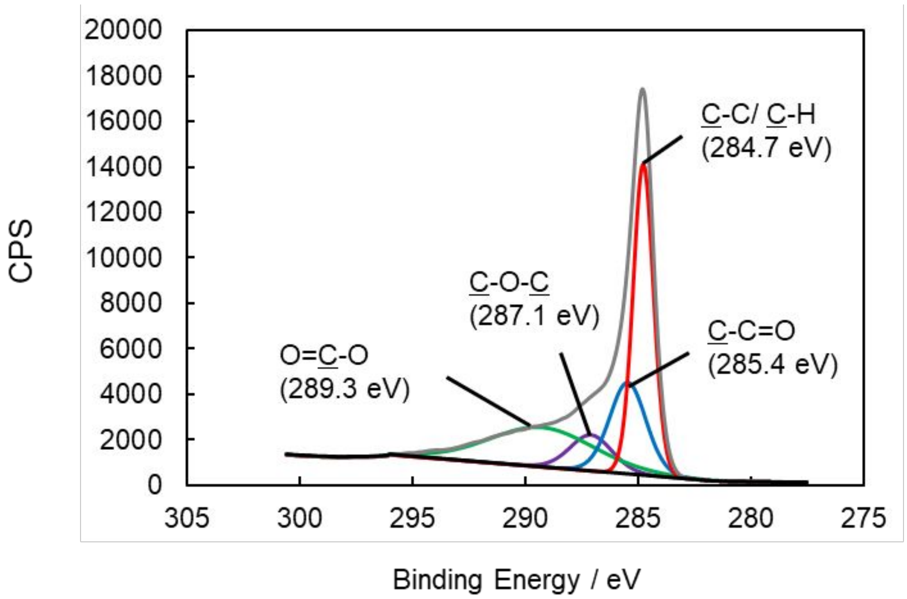

Figure S6. XPS C1s spectrum of GMgOC with peak deconvolution. Four components are identified at 284.7 eV (C–C/C–H), 285.4 eV (C–C=O), 287.1 eV (C–O–C-containing species), and 289.3 eV (O=C–O).

Section S7. Enrichment Factor and Mechanism Analysis

The solution-phase calibration curve was obtained by adding acetone at concentrations of 0, 0.05, 0.1, 0.3, 0.5, 0.7, 1.0, 1.5, and 2.0 mM to 0.1 M phosphate buffer (pH 7.0) containing 10 mM 1-methoxy PMS and 1 mM NADH, and measuring the chronoamperometric response at −0.4 V. Figure S7 shows the solution-phase calibration curve.

A concentration-dependent response was confirmed in the solution phase over the range

0.05–2.0 mM. The apparent Michaelis–Menten constant (Km) in the solution phase was calculated to be 19,193 ppb (0.33 mM).

Comparison of the apparent Km values in the solution phase (19,193 ppb) and the gas phase (217 ppb) yields an approximate enrichment factor (EF) of ~88 (= 19,193 / 217). This substantial reduction in the apparent Km in the gas-phase measurement is tentatively attributed to the adsorption and preconcentration of acetone within the porous GMgOC electrode structure, which increases the local analyte concentration at the enzyme–electrode interface relative to the bulk gas-phase concentration. It should be noted that this is an approximate comparison between solution-phase and gas-phase K_m values obtained under different experimental conditions (dissolved acetone vs. acetone vapor), and the two values are not directly equivalent. A rigorous, systematic preconcentration analysis—including measurement of mass-transfer coefficients, gas–liquid partition coefficients, and pore-diffusion modeling—was not conducted in this study and is planned as future work.

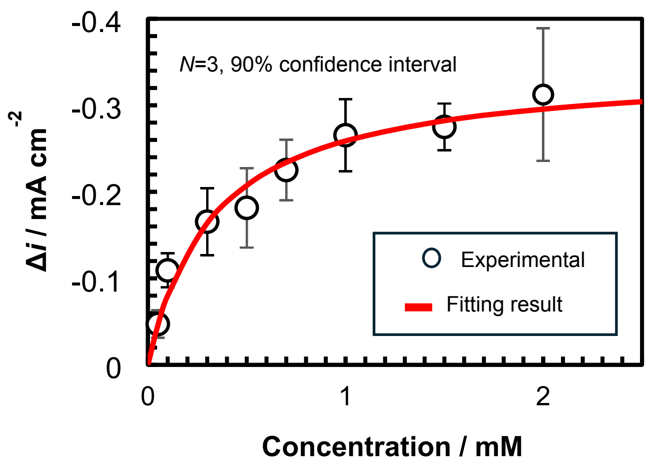

Figure S7. Solution-phase calibration curve: current density vs. acetone concentration (0.05–2.0 mM) in 0.1 M phosphate buffer (pH 7.0) containing 10 mM 1-methoxy PMS and 1 mM NADH. Measurement potential: −0.4 V, measurement time: 500 s.

Section S8. Selection Rationale for s-ADH

s-ADH was selected as the sensing enzyme because of its substrate specificity toward secondary alcohols and their ketone counterparts (acetone and 2-propanol), which provides selectivity over primary alcohols such as ethanol. The reversible nature of the

enzymatic reaction allows operation in either acetone-detection mode (reduction, pH 5.5–7.5) or 2-propanol-detection mode (oxidation, pH 8.0–9.5) [6–8]. The present sensor operates in the acetone-reduction mode at pH 7.0.

The selectivity of s-ADH toward secondary alcohols over primary alcohols has been demonstrated in prior biosniffer studies. Chien et al.[6] reported that an s-ADH-based biosniffer showed only a minor cross-response to 2-propanol and negligible responses to other common VOC interferents (ethanol, acetaldehyde, isoprene). However, a direct selectivity study under the present electrochemical sensor configuration was not performed in this study, which is acknowledged as a limitation. Future work will include systematic selectivity evaluation against common skin gas components.

Section S9. Calculation of Limit of Detection (LOD) and Limit of Quantification (LOQ)

The limit of detection (LOD) and limit of quantification (LOQ) were calculated from the slope of the linear calibration curve and the standard deviation of the background current as follows:

$$\mathrm{LOD} = 3\sigma / S \qquad \text{(S2)}$$

$$\mathrm{LOQ} = 10\sigma / S \qquad \text{(S3)}$$

where σ is the standard deviation of the background current density measured over 500 s at −0.4 V in the absence of acetone (0 ppb), and S is the slope of the linear calibration curve in the low-concentration range (50–100 ppb).

The calculated values are: LOD = 75 ppb and LOQ = 250 ppb.

The linear calibration curve in the range 50–100 ppb had a slope $S = 0.0028$ mA cm^{-2} ppb^{-1} ($R^2 \geq 0.99$). Above 100 ppb, the response deviates from linearity, consistent with partial saturation of the enzyme-accessible sites within the GMgOC pore structure. The LOD of 75 ppb is below the reported skin gas acetone concentration in healthy adults (77–97 ppb, ref 14 of the main manuscript), confirming the sensor's capability for skin gas detection.

Section S10. Sensor Selectivity

The selectivity of the present sensor depends on the substrate specificity of the immobilized enzyme s-ADH. In prior studies using biosniffer-type sensors, s-ADH has been shown to selectively respond to secondary alcohols and their corresponding ketones (acetone and 2-propanol), with negligible reactivity toward primary alcohols such as ethanol[6].

However, a direct selectivity evaluation under the present electrochemical sensor

configuration—such as exposure to ethanol, acetaldehyde, isoprene, or other common skin gas components—was not performed in this study. Selectivity inference is therefore based solely on the reported biochemical substrate specificity of s-ADH from the literature[6]. A systematic selectivity study, including interference testing with relevant VOCs, is planned as future work.

Section S11. Scope of the Present Study and Future Work

The present study constitutes a proof-of-concept demonstration of a screen-printed enzymatic electrochemical acetone gas sensor on a porous polyimide substrate. The following aspects were not evaluated in this study and are planned as future work:

Selectivity: Direct evaluation against common skin gas interferents (ethanol, acetaldehyde, isoprene, 2-propanol) using controlled gas mixtures.

Raman spectroscopy: D-band/G-band characterization of GMgOC to quantify the degree of graphitization and the effect of GMA grafting on carbon structure.

Electrochemical impedance spectroscopy (EIS): Step-by-step impedance characterization to confirm enzyme loading and electrolyte encapsulation effects on charge-transfer resistance.

Long-term stability: Systematic evaluation of signal retention over days to weeks under storage and operational conditions.

Reproducibility (inter-sensor RSD): Evaluation of fabrication repeatability across independently prepared sensors.

Response and recovery time: Formal measurement of t_{a0} response time and recovery time (baseline restoration after analyte removal); investigation of memory effects and mitigation strategies.

NADH cofactor stability: Evaluation of NADH stability in the enclosed electrolyte over extended storage periods; development of NADH replenishment or stabilized electrolyte strategies.

Temperature and humidity dependence: Assessment of sensor performance under varying ambient temperature and relative humidity.

Performance in air atmosphere: Evaluation of the effect of background gas composition (O_2, H_2O, CO_2) on sensor response.

Contact angle measurement: Quantitative characterization of liquid-blocking performance of the porous polyimide substrate.

GC–MS validation: Real sample measurements (skin gas from volunteers) with parallel GC–MS reference analysis.

Power consumption: Quantitative measurement of total energy consumption under

realistic wearable-device operating conditions.
BET surface area: Measurement of BET surface area and pore size distribution of MgOC and GMgOC.
Comparison of MgOC and GMgOC: Electrochemical and structural comparison of unmodified MgOC and GMA-grafted GMgOC electrodes.

References

(1) Tokyo Ohka Kogyo Co., Ltd. Product page for PIM-1000N. https://www.tok.co.jp/products/newfield/list/microprocessingfilm/porous

(2) Gastec Corporation. Permeater technology. https://www.gastec.co.jp/technology/knowledge/permeater/

(3) Shitanda, I.; Kato, T.; Suzuki, R.; Aikawa, T.; Hoshi, Y.; Itagaki, M.; Tsujimura, S. Stable Immobilization of Enzyme on Pendant Glycidyl Group-Modified Mesoporous Carbon by Graft Polymerization of Poly(Glycidyl Methacrylate). Bull. Chem. Soc. Jpn. 2020, 93, 32–36.

(4) Suzuki, R.; Shitanda, I.; Aikawa, T.; Tojo, T.; Kondo, T.; Tsujimura, S.; Itagaki, M.; Yuasa, M. Wearable Glucose/Oxygen Biofuel Cell Fabricated Using Modified Aminoferrocene and Flavin Adenine Dinucleotide-Dependent Glucose Dehydrogenase on Poly(Glycidyl Methacrylate)-Grafted MgO-Templated Carbon. J. Power Sources 2020, 479, 228807.

(5) Barbey, R.; Laporte, V.; Alnabulsi, S.; Klok, H. Macromolecules 2013, 46, 6151.

(6) Chien, P.-J.; Suzuki, T.; Tsujii, M.; Ye, M.; Minami, I.; Toda, K.; Otsuka, H.; Toma, K.; Arakawa, T.; Araki, K.; Iwasaki, Y.; Shinada, K.; Ogawa, Y.; Mitsubayashi, K. Biochemical Gas Sensors (Biosniffers) Using Forward and Reverse Reactions of Secondary Alcohol Dehydrogenase for Breath Isopropanol and Acetone as Potential Volatile Biomarkers of Diabetes Mellitus. Anal. Chem. 2017, 89, 12261–12268.

(7) Ye, M.; Chien, P.-J.; Toma, K.; Arakawa, T.; Mitsubayashi, K. An Acetone Bio-sniffer (Gas Phase Biosensor) Enabling Assessment of Lipid Metabolism from Exhaled Breath. Biosens. Bioelectron. 2015, 73, 208–213.

(8) Toma, K.; Tsujii, M.; Arakawa, T.; Iwasaki, Y.; Mitsubayashi, K. Dual-Target Gas-Phase Biosensor (Bio-sniffer) for Assessment of Lipid Metabolism from Breath Acetone and Isopropanol. Sens. Actuators B 2021, 329, 129260.
